# Antiplatelet Therapy in High-Bleeding Risk Patients Undergoing PCI: Walking a Tightrope

**DOI:** 10.31083/j.rcm2306207

**Published:** 2022-06-01

**Authors:** Davis Jones, Johny Nicolas, Frans Beerkens, Mohan Satish, Daniel Feldman, Davide Cao, Alessando Spirito, Roxana Mehran

**Affiliations:** ^1^The Zena and Michael A. Wiener Cardiovascular Institute, Icahn School of Medicine at Mount Sinai, NY 10029-6574, US; ^2^Cardiovascular Department, Humanitas Gavazzeni, 24125 Bergamo, Italy

**Keywords:** high bleeding risk, HBR, percutaneous coronary intervention, antiplatelet therapy, antithrombotic therapy, DAPT

## Abstract

Historically, prevention from ischemic events with dual antiplatelet therapy (DAPT) post percutaneous coronary intervention (PCI) took precedence over protection from bleeding. However, increasing data suggest that major bleeding complications are as detrimental as ischemic events. Awareness about the prognostic impact of bleeding prompted the search for new strategies aimed at maximizing both ischemic and bleeding protection. This is noteworthy because patients at high bleeding risk (HBR) have generally been underrepresented in clinical trials on DAPT and they often are at increased risk of ischemic events as well. The present review discusses the evidence base for new pharmacotherapeutic strategies to decrease bleeding risk without compromising ischemic protection among HBR patients undergoing PCI, including shortening DAPT duration, early aspirin withdrawal, and ${\mathrm{P2Y}}_{12}$ inhibitor de-escalation.

## 1. Introduction

Antiplatelet agents constitute the foundation therapy for secondary prevention of thromboembolic events after percutaneous coronary intervention (PCI) with drug-eluting stent (DES) [1]. Guidelines currently recommend, the use of dual antiplatelet therapy (DAPT), a combination of aspirin and a ${\mathrm{P2Y}}_{12}$ receptor inhibitor, following DES implantation for at least 6 months in patients with stable coronary artery disease (CAD) and 12 months in cases of acute coronary syndrome (ACS) [2, 3]. Historically, prevention of ischemic events and ST elevations took precedence over protection from bleeding, leading to studies exploring DAPT regimens greater than 12 months [4]. Recently, several studies revealed that major bleeding complications related to prolonged DAPT carry a similar prognostic impact as ischemic events [5, 6, 7]. Furthermore, the introduction of newer generation DES with thinner struts and more biocompatible polymers decreased the risk of stent-related adverse events thus providing a rationale for shorter DAPT regimens. Therefore, an antithrombotic therapy strategy that mitigates the bleeding risk while maintaining ischemic protection seems most desirable in contemporary PCI practice. Balancing the ischemic and bleeding risks becomes even more challenging in patients with multiple comorbidities and particularly those at high bleeding risk (HBR). There is a scarcity of data regarding the optimal antiplatelet strategy in HBR patients as they were either excluded or underrepresented in most randomized controlled trials (RCT) that shaped contemporary guidelines [8, 9]. Aiming at decreasing the bleeding complications associated with prolonged DAPT, especially among HBR patients, several studies investigated novel antithrombotic therapy strategies that shorten DAPT duration or decrease its intensity over time. In this review, we aim to define HBR patients, discuss bleeding risk stratification tools, and review recent advances in post-PCI pharmacotherapy.

### 1.1 High-Bleeding Risk Patients

Advancements in PCI technologies have allowed extending this treatment option to high-risk patients who were traditionally managed conservatively. These patients typically have extensive CAD and multiple comorbidities that, not only increase their risk for thromboembolic events, but also for bleeding complications. Indeed, a study of an all-comer population undergoing DES implantation has shown that as high as 1 in every 15 patients experienced post-discharge bleeding at a median of 300 days after the procedure [5]. Interestingly, the impact of bleeding on two-year mortality was significantly larger compared with post-discharge MI [5]. This study, together with other observational studies, shed light on the prognostic relevance of post-discharge bleeding after PCI [10].

**Fig. 1.**
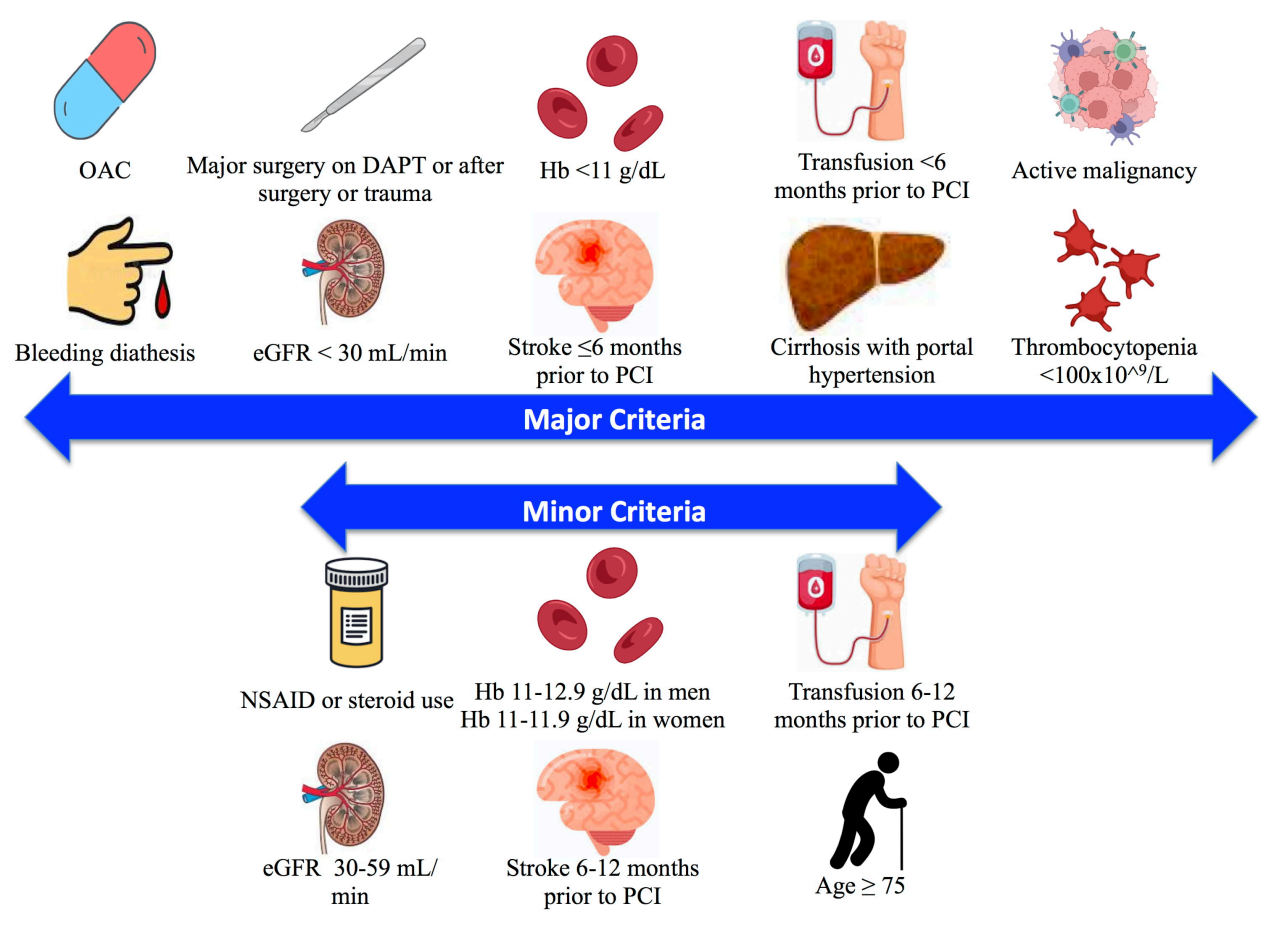
**ARC-HBR definition of HBR**. Major and minor risk factors used in the definition for HBR [11]. 1 major or ≥2 minor criteria qualify as HBR. DAPT, dual anti-platelet therapy; eGFR, estimated glomerular filtration rate; Hb, hemoglobin; NSAID, non-steroidal anti-inflammatory drugs; OAC, oral anti-coagulant.

Finding patients at HBR is of highest importance for the management of antithrombotic therapy after PCI. Nonetheless, a lack of standardization in defining this population limits the generalizability of trial results as well as clinical decision-making. Based on review of the literature, the Academic Research Consortium (ARC) recently published an agreement definition of patients at HBR based on fulfillment of specific criteria (Fig. 1, Ref. [11]) [12]. Several registry-based studies have validated the ARC-HBR definition by showing an incidence of Bleeding Academic Research Consortium (BARC) 3 or 5 bleeding risk of ≥4% in HBR patients at one year after PCI [13, 14, 15]. Moreover, these studies revealed that HBR patients account for around a third of all PCI patients, reiterating the need for tailored antiplatelet therapy strategies that mitigate the bleeding risk.

### 1.2 Contemporary Bleeding Risk Scores 

Over the past years, numerous risk scores have been designed to inform and guide decision making on DAPT duration and intensity after PCI. The European Society of Cardiology guidelines recommend using the DAPT and PRECISE-DAPT risk scores [16, 17]. The DAPT scoring system was developed using predictors of both ischemic and bleeding events to identify patients who derive the greatest benefit over harm from prolonging DAPT beyond 12 months of PCI [18]. Conversely, the PRECISE-DAPT was developed to assess the risk of out of hospital bleeding up to 2 years post-PCI [19]. The PARIS score encompasses two separate prediction models to evaluate ischemic and bleeding risks after PCI [20]. Although these scores share similar components, each has its own features (Table 1). The DAPT score included somewhat lower risk patients who were event-free at 12 months post-PCI, while both PRECISE-DAPT and PARIS included patients immediately after discharge of index PCI. While HBR patients composed approximately 25% of all subjects considered, each scoring system identified different rates of bleeding ranging from 1% to 10% [11]. Therefore, it has been difficult to confidently use such scoring systems in HBR patients undergoing PCI. Recently, Urban *et al*. [12] developed the ARC-HBR trade off model, which predicts the risk of non-periprocedural major bleeding and thrombotic events at one year among HBR patients who have undergone PCI. Although this was the first risk score especially dedicated to HBR patients, it should be noted that this tool was derived from studies using different DAPT durations (i.e., driven by the protocol of the study or guideline-based) and recommendations should not be solely made based on its risk predictions.

**Table 1.** **Various bleeding risk scores**.

	REACH-39	DUTCH ASA Score37	DAPT41	PARIS38	PRECISE-DAPT32	BleeMACS36
Year	2010	2014	2016	2016	2017	2018
Database	REACH	Dutch ASA registry	DAPT randomized trial	PARIS	8 randomized trials	BleeMACS
Number	56616	235531	11648	4190	14963	15401
Population	Risk of atherosclerosis	New low-dose ASA	Post-PCI patients event free 12 mo after index	All PCI	Patients undergoing PCI	ACS undergoing PCI
Definition	Non-fatal hemmorhage or bleeding leading to both hospitalization and transfusion at 2 years	UGIB at median follow up 530 d	GUSTO moderate or severe bleeding	BARC 3 or 5 after 2 y	TIMI major or minor with median follow up 552 d	Intracranial bleed or any bleed requiring hospitalization or transfusion at 1 year
Bleeding risk score factors	Age, PAD, CHF, DM, HLD, HTN, Smoking, Anti-platelet, OAC	Age, Anemia, DM, Other anti-platelet, OAC	Age, PAD, HTN, Renal insuffiency	Age, BMI, Anemia, Triple therapy, Smoking, Renal insuffiency	Age, Previous bleed, WBC, Hb, Cr clearance	Age, HTN, PAD, Prior bleed, Malignancy, Cr clearance, Hb
Validation discrimination	AUC 0.68	AUC 0.64	AUC 0.68	AUC 0.72	AUC 0.73	AUC 0.71
External validation	CHARISMA	Dutch Health Insurance Database	PROTECT	ADAPT-DES	PLATO and BernPCI Registry	SWEDEHEART
External validation discrimination	AUC 0.64	AUC 0.63	AUC 0.64	AUC 0.64	AUC 0.70 and 0.66	AUC 0.65

ASA, aspirin; AUC, area under curve; BARC, Bleeding Academic Research Consortium; BMI, body mass index; CHF, congestive heart failure; Cr, creatinine; DAPT, dual antiplatelet therapy; DM, diabetes mellitus; GUSTO, global utilization of streptokinase and TPA for occluded arteries; Hb, hemoglobin; HTN, hypertension; HLD, hyperlipidemia; OAC, oral anticoagulant; PAD, peripheral artery disease; PCI, percutaneous coronary intervention; TIMI, thrombolysis in myocardial infarction; UGIB, upper gastrointestinal bleed; WBC, white blood cell.

## 2. Anti-Thrombotic Strategies 

### 2.1 Shortening DAPT Duration

The paradigm in interventional cardiology research has shifted over the past few years into testing strategies that unite modern DES platforms with short DAPT durations (Table 2, Ref. [21, 22, 23, 24, 25]). LEADERS-FREE was a randomized double blind trial comparing outcomes of HBR patients receiving the polymer-free biolimus-eluting BioFreedom stent vs. a similar bare metal stent (BMS); patients in both arms were maintained on DAPT for one month after PCI [21]. The BioFreedom stent was found to be superior to BMS with regards to the composite of cardiac death, MI, or stent thrombosis, largely driven by decreased rates of MI. Conversely, the ONYX ONE trial examined the same BioFreedom stent in comparison to the durable-polymer zotralimus-eluting Resolute Onyx stent in a similar HBR population [22]. The Resolute Onyx stent was found to be non-inferior to the BioFreedom stent with respect to the same primary outcome as above. However, since both the LEADERS-FREE and ONYX ONE trials preceded the ARC-HBR consensus, definitions of HBR differed, making comparisons among studies difficult to interpret. Most notably, LEADERS-FREE and ONYX ONE considered age ≥75 alone as HBR criterion for study inclusion. Despite this, improvements in stent technologies have clear benefits in patients at HBR, most notably in the ability to decrease DAPT duration. The SENIOR trial examined outcomes of elderly patients ≥75 years old by randomizing the bioabsorbable-polymer everolimus-eluting Synergy stent vs. BMS followed by shortened DAPT (1 month in stable patients and 6 months if unstable). DES was superior regarding the primary outcome of all-cause death, MI, stroke, or ischemia driven target vessel revascularization at one year, mostly driven by the latter [23]. Additionally, the DEBUT trial examined whether drug-coated stents were non-inferior to BMS in HBR patients [26]. Not only did they determine that drug-eluting stents were non-inferior to BMS with respect to major adverse cardiac events (MACE) after 9 months, they found superiority. Although these 4 studies showed that shortening DAPT duration is a safe and effective strategy in HBR patients who undergo PCI with new-generation DES, optimal therapy durations cannot be determined based on these trial designs.

**Table 2.** **Trials of devices with short DAPT**.

Trial	N	Population	DAPT	Intervention	Control	Primary outcome	Result
LEADERS-FREE [21]	2466	CAD requiring PCI	1 month	BioFreedom DCS	BMS	1: Cardiac death, MI, or stent thrombosis	1: 9.4% vs. 12.9%, HR 0.71, 95% CI 0.56–0.91, *p *< 0.001
						2: TLR	2: 5.1% vs. 9.8%, HR 0.5, 95% CI 0.37–0.69, *p *< 0.001
SENIOR [23]	1200	≥75 yo, stable angina, or ACS	1 month stable CAD, 6 months ACS	Synergy DES	BMS	MACCE at 1 year	12% vs. 16%, RR 0.71, 95% CI 0.52–0.94, *p* = 0.02
ONYX-ONE [22]	1996	HBR patients undergoing PCI	1 month	Resolute Onyx DES	BioFreedom DCS	Non-inferiority for cardiac death, TVMI, TLR at 1 year	17.1% vs. 16.9%, *p* = 0.01
EVOLVE Short DAPT [25]	2009	HBR patients with stable or unstable angina	3 months	SYNERGY DES	DES	1: Death or MI	1: 5.6% vs. 5.7%, *p* = 0.0016 non-inferiority
						2: stent thrombosis	2: 0.2%, *p* = 0.0005 for comparison to 1% performance goal
XIENCE 28 [24]	1392	HBR patients undergoing PCI	1 month	XIENCE DES	DES	1: Death or MI between 1 and 6 months	1: 3.5% vs. 4.2%, *p *< 0.0005 non-inferiority
						2: BARC 2,3,5 bleeding between 1 and 6 months	2: 4.9% vs. 5.9%, *p* = 0.19
XIENCE 90 [24]	1693	HBR patients undergoing PCI	3 months	XIENCE DES	DES	1: Death or MI between 3 and 12 months	1: 5.4% vs. 5.4%, *p *< 0.0063 non-inferiority
						2: BARC 2,3,5 bleeding between 3 and 12 months	2: 5.1% vs. 7.0%, *p* = 0.0687
						3. Stent thrombosis between 3 and 12 months	3: 0.2%, *p *< 0.0001 for 1.2% performance goal

ACS, acute coronary syndrome; BARC, Bleeding Academic Research Consortium; BMS, bare metal stent; CAD, coronary artery disease; CI, confidence interval; DAPT, dual anti-platelet therapy; DCS, drug coated stent; DES, drug-eluting stent; HBR, high bleeding risk; HR, hazard ratio; MACCE, major adverse cardiac and cerebrovascular events; MI, myocardial infarction; N, Number of patients; PCI, percutaneous coronary intervention; RR, relative risk; TLR, target lesion revascularization; TVMI, target vessel myocardial infarction.

More recently, the EVOLVE Short DAPT registry enrolled n = 1437 HBR patients treated with the Synergy stent followed by 3-month DAPT [25]. Such short DAPT duration was found to be non-inferior to a historical cohort of patients treated with 12-month DAPT with respect to death or MI but failed to show and advantage in terms of bleeding. Notably, the study was non-randomized and the control group was not uniform as it included multiple different stent types, potentially limiting the generalizability of the results. The XIENCE Short DAPT program included 3 registries (XIENCE 28 Global and 28 USA, and XIENCE 90) for a total of n = 3652 HBR patients undergoing PCI with the fluoropolymer-based cobalt-chromium Everolimus-eluting Xience stent who discontinued DAPT at 1 or 3 months post-PCI if event-free and treatment-adherent [27]. Both short DAPT regimens (1 and 3 months) were non-inferior to standard DAPT (6 to 12 months) with respect to death or MI and superior with respect to major bleeding, after propensity score-stratification vs. an historical group of patients receiving the same stent [24]. In a subsequent exploratory analysis from the XIENCE data, 1 month of DAPT was shown to have comparable ischemic outcomes and lower bleeding risk compared with 3-month DAPT [28]. MASTER DAPT was the first RCT testing different DAPT durations in HBR patients treated with a new-generation DES. The trial included 4434 HBR patients who underwent placement of the biodegrable-polymer sirolimus-eluting Ultimaster stent [29]. Subjects who were event free after 1 month of index PCI were either randomized to DAPT discontinuation followed by either aspirin or a ${\mathrm{P2Y}}_{12}$ inhibitory monotherapy or continuation of DAPT for at least 5 additional months. The short DAPT (1 month) regimen was shown to be non-inferior to prolonged DAPT with regard to net adverse clinical events and MACE [29]. Specifically, the composite of major or clinically relevant bleeding was observed in 6.5% in the experimental group as compared to 9.4% in the control without tradeoff in ischemic events. Therefore, shortening DAPT duration to 1 to 3 months may be a reasonable approach in selected HBR patients, pending additional data from large clinical trials.

### 2.2 ${\mathrm{P2Y}}_{12}$ Inhibitor Monotherapy

Aspirin has been the mainstay therapy for long-term secondary prevention of ischemic events for decades. Recently, its undisputed benefits have been challenged for several reasons: (1) increased risk of intracranial and extracranial bleeding, especially in HBR patients, (2) widespread use of optimal medical therapy including disease-modifying drugs (i.e., angiotensin converting enzyme-inhibitors, angiotensin receptor blockers, statins, etc.), and (3) the introduction of more potent antiplatelet agents. However, the introduction of new antiplatelet agents in PCI practice has always requested these new agents to prove their benefits on a background of aspirin therapy such that their individual effects have never been truly assessed. The PLATO trial showed that the more potent ${\mathrm{P2Y}}_{12}$ inhibitor, ticagrelor, is superior to clopidogrel in reducing ischemic events at 12 months among ACS patients, although at the cost of increased bleeding [30]. To note, PLATO also suggested that low dose (<300 mg) aspirin was more effective than a high dose (≥300 mg) in preventing ischemic events when combined with ticagrelor [31]. This raised the question as to whether aspirin is at all needed in presence of potent ${\mathrm{P2Y}}_{12}$ inhibitors [32, 33]. The GLOBAL LEADERS trial addressed this question in an all-comer population of patients undergoing PCI for stable CAD or ACS [34]. It randomized over 15,000 patients to either ticagrelor monotherapy for 23 months after 1 month of DAPT or 12 months of DAPT followed by aspirin monotherapy. Ticagrelor monotherapy was not superior to 12-month DAPT for the primary endpoint of all-cause death and new Q-wave MI (3.81% in experimental vs. 4.37% in control; *p* = 0.073) and was associated with similar rates of bleeding events [34]. The study had several limitations including an open label design [35]. GLASSY, a GLOBAL LEADERS adjudication substudy conducted at the top-10 enrolling sites (n = 7585) yielded similar conclusions as the parent trial but for the first time suggested a reduction in thrombotic events (MI and ST) with ticagrelor monotherapy vs. aspirin between 1 and 2 years post-PCI [36]. The SMART CHOICE study showed ${\mathrm{P2Y}}_{12}$ inhibitor monotherapy after 3-month DAPT was non-inferior to the standard treatment of 12 months with respect to major adverse cardiac and cerebrovascular events (MACCE) a composite of all cause death, stroke, MI [37]. In line with previous studies, they found a decrease in bleeding rates, specifically bleeding BARC 2-5, but did not find any differences in major bleeding [37]. Unlike the GLOBAL LEADERS trial, SMART CHOICE included multiple ${\mathrm{P2Y}}_{12}$ inhibitors with clopidogrel the most frequently used, giving evidence to its benefit as a monotherapy, similar to STOPDAPT2. Although this study was conducted in a low-risk Asian population, decreasing the bleeding risks would most certainly be beneficial to those at HBR. Similar results were found in the STOPDAPT-2 trial where they found that 1 month of DAPT followed by clopidogrel monotherapy was superior to 12 months of DAPT in a composite of cardiovascular and bleeding events, largely driven by a reduction in bleeding [38]. However, the trial included mainly low-risk Japanese patients with very high rates of intravascular imaging use, therefore its generalizability has been questioned. More recently, the STOPDAPT-2 ACS trial, an extension of STOPDAPT-2, enrolling only ACS patients, showed that 1-month DAPT followed by clopidogrel monotherapy was not non-inferior with respect to net adverse events (including ischemic and bleeding endpoints) when compared to standard DAPT [39]. These results were driven by a significant decrease in the occurrence of major bleeding events, which was offset by a concomitant increase in ischemic events. The TICO trial further investigated ticagrelor monotherapy compared to DAPT among patients with ACS. Although event rates were lower than expected, ticagrelor monotherapy after 3 months of DAPT decreased the incidence of net adverse clinical events (composite of major bleeding and adverse cardiac and cerebrovascular events) [40]. Again, this difference was mostly driven by a decrease in bleeding complications, with no tradeoff in ischemic events. Even though this study excluded patients at HBR, it further supported the use of ${\mathrm{P2Y}}_{12}$ inhibitor monotherapy, reducing overall risks of bleeding.

**Fig. 2.**
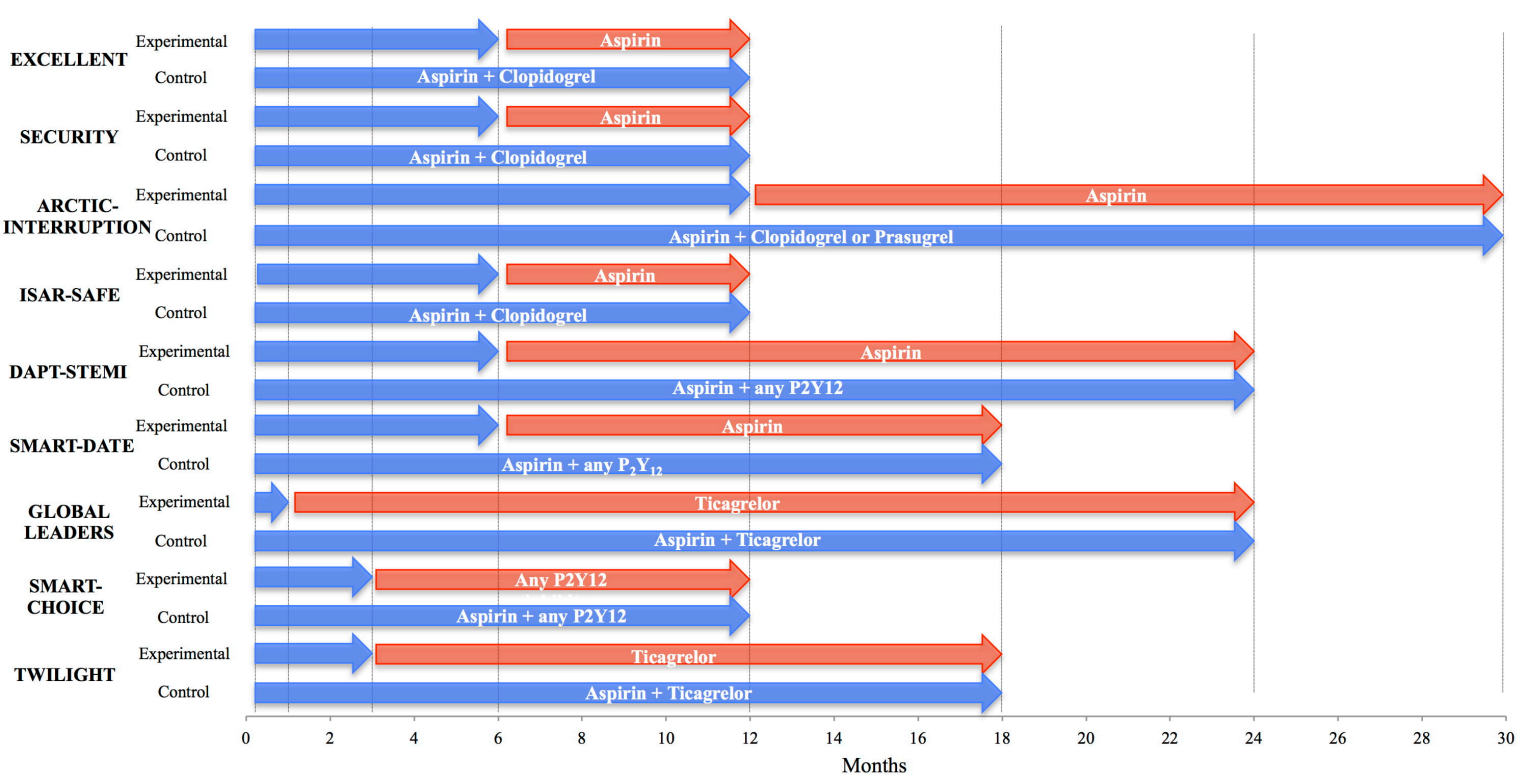
**Trial designs of shortening DAPT after PCI regardless of stent design**. Comparison of studies of short DAPT duration regardless of specific stent designs [34, 37, 41, 46, 47, 48, 49, 50, 51]. Blue arrows indicate duration of DAPT, red arrows indicate duration of monotherapy. DAPT, dual antiplatelet therapy.

**Fig. 3.**
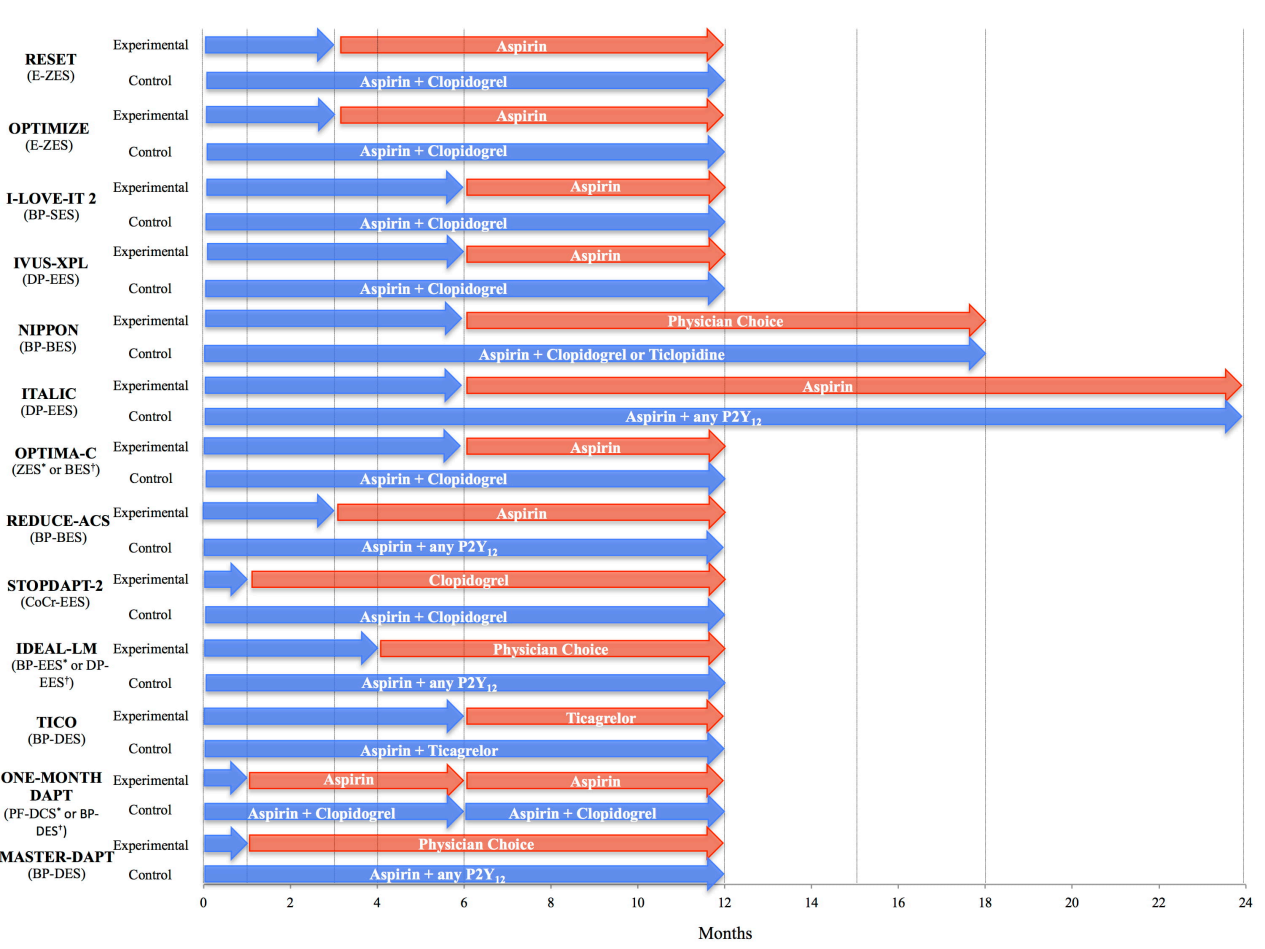
**Short DAPT trial with specific stent designs**. Comparison of studies with short DAPT designs with specific stent designs [29, 38, 40, 52, 53, 54, 55, 56, 57, 58, 59, 60, 61]. Blue arrows indicate duration of DAPT, red arrows indicate duration of monotherapy. BES, biolimus eluting stent; BP-BES, bioresorbable polymer biolimus-eluting stent; BP-DES, bioresorbable polymer drug-eluting stent; BP-EES, bioresorbable polymer everolimus-eluting stent; BP-SES, bioresorbable-polymer sirolimus-eluting stent; CoCr-EES, cobalt chromium everolimus-eluting stent; DAPT, dual antiplatelet therapy; DP-EES, durable polymer everolimus-eluting stent; E-ZES, endeavor zotarolimus-eluting stent; PF-DCS, polymer free drug coated stent; ZES, zotarolimus-eluting stent. * indicates experimental group. † indicates control group.

The TWILIGHT study examined ticagrelor monotherapy following 3-month DAPT in high-risk patients undergoing PCI. Patients were considered at high risk for ischemic and bleeding events if they fulfilled at least one clinical and one angiographic high-risk feature. This double-blinded placebo-controlled study randomized patients to receive either ticagrelor monotherapy or ticagrelor plus aspirin for 12 months after being event free for 3 months post PCI. Ticagrelor monotherapy was shown to reduce the incidence of the primary endpoint of BARC 2, 3, or 5 bleeding without an increase in ischemic events [41]. A sub-analysis of the TWILIGHT trial looking at patients who qualify as HBR based on ARC-HBR criteria showed consistent results, with larger absolute risk reduction in major bleeding observed with ticagrelor monotherapy in HBR versus non-HBR patients [42]. Several meta-analyses of the above studies showed decreased risks of bleeding, while no concomitant increase in events [43, 44, 45]. Although many of these studies do not specifically examine HBR patients, a short DAPT duration followed by ${\mathrm{P2Y}}_{12}$ inhibitor monotherapy emerged as a safe and effective bleeding-avoidance strategy, although extra caution might be needed in those presenting with ACS (Table 3, Figs. 2,3, Ref. [29, 34, 37, 38, 40, 41, 46, 47, 48, 49, 50, 51, 52, 53, 54, 55, 56, 57, 58, 59, 60, 61]).

**Table 3.** **Trials including anti-platelet monotherapies**.

Trial	N	Population	Major inclusion and exclusion criteria	Intervention	Control	Primary outcome	Result
GLOBAL LEADERS [34]	15968	Stable CAD or ACS with biolimus A9-eluting stent	**Inclusion:** 50% of more stenosis in ≥1 coronary	ASA + ticagrelor for 1 month followed by 23 months ticagrelor monotherapy	DAPT for 12 months followed by ASA monotherapy	Composite of all-cause mortality or non-fatal new Q-wave MI at 2 years	3.81% vs. 4.37%, RR 0.87, 95% CI 0.75–1.01, *p* = 0.073
			**Exclusion:** Chronic oral anti-coagulation				
STOPDAPT-2 [38]	3045	PCI	**Inclusion:** PCI with CoCr-EES stent without complications post PCI	1 month DAPT followed by clopidogrel monotherapy	DAPT	Composite of CV death, MI, stroke, stent thrombosis, major or minor bleeding at 1 year	2.36% vs. 3.70%, HR 0.64, 95% CI 0.42–0.98, *p *< 0.01 non-inferiority, and *p* = 0.04 superiority
			**Exclusion:** Need for oral anticoagulation, history of intracranial bleeding				
SMART-CHOICE [37]	2993	PCI with DES placement	**Inclusion:** 50% or more stenosis in ≥1 coronary	DAPT for 3 months followed by monotherapy	DAPT	Composite of death, MI, stroke at 1 year	2.9% vs. 2.5%, one-sided 95% CI -∞-1.3%, *p* = 0.007 non-inferiority
			**Exclusion:** Cardiogenic shock, active bleeding				
TWILIGHT [41]	7119	High risk for bleeding or ischemia undergoing PCI	**Inclusion:** 1 clinical feature and one angiographic feature with high risk ischemia or bleeding events	3 months DAPT followed by monotherapy	DAPT	1: BARC 2,3 or 5 bleeding at 1 year	1: 4.0% vs. 7.1%, HR 0.56, 95% CI 0.45–0.68, *p *< 0.00
			**Exclusion:** STEMI, cardiogenic shock, oral anticoagulation			2: Composite of death, MI, stroke	2: 3.9% vs. 3.9%, HR 0.99, 95% CI 0.78–1.25, *p *< 0.001 non-inferiority
TICO [40]	3056	ACS requiring PCI	**Inclusion:** PCI with Orsiro stent for ACS	Ticagrelor monotherapy after 3 months DAPT	DAPT	Composite of major bleeding, death, MI, stent thrombosis, stroke, or TVR at 1 year	3.9% vs. 5.9%, HR 0.66, 95% CI 0.48–0.92, *p* = 0.01
			**Exclusion:** prior hemorrhagic stroke, internal bleeding in last 6 weeks, hemoglobin ≤8 g/dL				
MASTER-DAPT [29]	4434	HBR receiving TANSEI DES	**Inclusion:**≥1 high bleeding risk criteria, PCI with TANSEI stent	1 month DAPT followed by ASA or ${\mathrm{P2Y}}_{12}$ monotherapy	DAPT	1: NACE	1: 7.5% vs. 7.7%, AD –0.23%, 95% CI –1.8–1.33, *p *< 0.001 non inferiority
			**Exclusion:** Treatment for ISR, BARC ≥2 bleeding			2: MACCE	2: 6.1% vs. 5.9%, AD 0.11%, 95% CI –1.29–1.51, *p* = 0.001 non-inferiority
						3: MCB at 12 months	3: 6.4% vs. 9.2%, AD –2.78%, 95% CI –4.37 to –1.20, *p *< 0.001 superiority

ACS, acute coronary syndrome; AD, absolute difference; ASA, aspirin; BARC, Bleeding Academic Research Consortium; BMS, bare metal stent; CAD, coronary artery disease; CoCr-EES; cobalt chromium everolimus-eluting stent; CI, confidence interval; CV, cardiovascular; DAPT, dual anti-platelet therapy; DES, drug-eluting stent; HBR, high bleeding risk; HR, hazard ratio; ISR, in-stent restenosis; MACCE, major adverse cardiac and cerebrovascular events; MCB, major or clinically relevant bleeding; MI, myocardial infarction; N, Number of patients; NACE, net adverse clinical events; PCI, percutaneous coronary intervention; RR, relative risk; TVR, target vessel revascularization.

### 2.3 DAPT Modulation by De-Escalation

#### 2.3.1 De-Escalation Tactics: Unguided

**Fig. 4.**
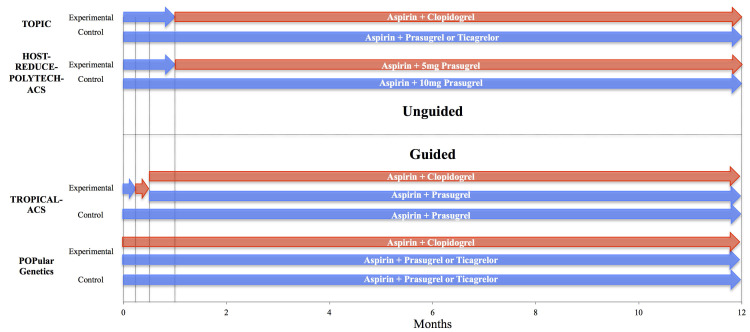
**Guided and Unguided de-escalation trials**. Comparison of trial designs of guided and unguided de-escalation strategies. Blue arrows indicate duration of potent therapies, red arrows indicate duration of less potent therapies (i.e., lower doses of prasugrel or ticagrelor vs. changing from prasugrel or ticagrelor to clopidogrel) [64, 65, 66, 67].

In patients presenting with ACS, guidelines recommend DAPT with a potent ${\mathrm{P2Y}}_{12}$ inhibitor (i.e., ticagrelor or prasugrel) and aspirin for about 12 months [62]. However, the benefits of potent ${\mathrm{P2Y}}_{12}$ inhibitors are mostly observed in the acute phase post PCI (i.e., the first 30 days) when the risk of ischemic events is highest. However, this risk decreases overtime while bleeding persists and proportional to the duration and intensity of antiplatelet therapies [63]. As a result, investigators have hypothesized that de-escalating therapy, such as switching to a less potent ${\mathrm{P2Y}}_{12}$ inhibitor or using a lower dose of the same agent after an initial course of potent DAPT, would mitigate bleeding risk without compromising patient safety (Fig. 4, Ref. [64, 65, 66, 67]). The TOPIC trial showed that patients with ACS had decreased risks of bleeding without increase in ischemic events when switching from a more potent ${\mathrm{P2Y}}_{12}$ inhibitor to clopidogrel one month after PCI [48]. This study, however, was limited by a small sample size (n = 646) with low protocol adherence. The HOST-REDUCE-POLYTECH-ACS trial showed similar results with decreasing dosages of prasugrel [65]. They found that decreasing the dose from 10 mg to 5 mg one month after PCI in ACS patients was associated with a significant decrease in net adverse clinical events, mainly driven by significant reductions in bleeding [65]. Although promising, the study findings may not be generalizable since the trial only included East Asian patients with different ischemic-bleeding risk profiles and a variable response to antiplatelet agents as compared with Western populations [68]. Prasugrel is however contraindicated in older (≥75) and lower body weight (<60 kg) patients who, therefore, had to be excluded from this trial [65, 69]. Since age greater than 75 is an HBR criterion, it may be difficult to extrapolate this data to HBR patients [11].

#### 2.3.2 De-Escalation Tactics: Guided

De-escalation of antiplatelet therapy can be guided by platelet function testing (PFT) or genetic testing. Although multiple modalities have been developed to determine platelet function, they all serve the purpose to determine how well platelets work to stop bleeding. They determine the residual ability of platelets to aggregate after doses of antiplatelet drug therapy Patients on ${\mathrm{P2Y}}_{12}$ inhibitors have decreased platelet reactivity; those with high platelet reactivity, despite the use of clopidogrel, should be maintained on more potent antiplatelet agents [70, 71]. As a result, it was thought that PFT could play a role in guiding escalation or de-escalation of therapy. The non-inferiority TROPICAL-ACS study randomized patients to standard therapy (12 months of DAPT with prasugrel) or de-escalation based on PFT results [66]. The experimental group received 1 week of prasugrel after discharge, followed by 1 week of clopidogrel. PFT was performed one week after initiation of clopidogrel, or 2 weeks post-discharge. If patients were found to have high platelet reactivity, they were escalated to prasugrel, otherwise were maintained on clopidogrel. The trial met non-inferiority with respect to net clinical benefit in the de-escalation group [66]. Although not statistically significant, the study observed a decrease in bleeding events in the de-escalation group compared with standard treatment.

The variable platelets reactivity to clopidogrel may lead to suboptimal antithrombotic protection [72]. Loss of function alleles, specifically the CYP2C19*2 and CYP2C19*3 alleles, have been identified as genetic causes for the decreased response to clopidogrel and its decreased efficacy [73, 74]. In those without this loss of function allele, clopidogrel was found to be equally effective as more potent ${\mathrm{P2Y}}_{12}$ inhibitors [75, 76]. The POPular Genetics study investigated if this gene identification could be utilized in guiding DAPT selection to decrease the risks of bleeding and ischemic events [67]. Patients were randomized to early genetic testing or standard DAPT with ticagrelor or prasugrel. Those with the loss of function allele were started on ticagrelor or prasugrel while non-carriers received clopidogrel. After 12 months, the genotype-guided group was non-inferior to standard treatment with regards to net adverse clinical events and major bleeding. A meta-analysis of 11 RCTs and 3 observational studies including 20,743 patients examined guided therapies compared to standard therapy, noting a significant decrease in the risk of MACE as well as lower rates of bleeding events though statistically non-significant [77]. However, the outcomes varied widely based on whether therapies were either escalated, resulting in decreased ischemic events and no increase in bleeding, or de-escalated, resulting in reduction in bleeding with no increase in ischemic events [77]. A network meta-analysis, which included 15 RCTs incorporating multiple different de-escalation strategies, demonstrated that DAPT de-escalation reduced risk of major or minor bleeding when compared to clopidogrel, ticagrelor, standard-dose prasugrel, and low dose prasugrel. There was also no change in composite of cardiovascular death, MI, and stroke [78]. Another study, including 5 RCTs of ACS-only patients corroborated these results with both guided and unguided de-escalation strategies [79]. These meta-analyses have all shown benefits of de-escalation, but as of now, no studies have examined differences among de-escalation therapies and ${\mathrm{P2Y}}_{12}$ inhibitor monotherapies, making it difficult to understand which strategy is preferred. Regardless, guided de-escalation therapies have clear benefits in preventing major bleeding, while preserving the safety of continued DAPT therapies (Table 4, Fig. 4, Ref [64, 65, 66, 67, 80]). However, PFT testing is not routinely performed or recommended as it has not shown a consistent clinical benefit [81]. Furthermore, genetic testing is not currently recommended either, as testing is expensive and there are many other factors that may play into the variability of clopidogrel’s effectiveness. Although there are currently no studies specifically aimed at examining de-escalation therapies in HBR patients, these therapies seem sensitive in this vulnerable population.

**Table 4.** **Trials of guided or unguided de-escalation strategies**.

Trial	N	Population	Major inclusion and exclusion criteria	Intervention	Control	Primary outcome	Result
TOPIC [64]	646	ACS requiring PCI	**Inclusion:** ACS, no adverse events at 1 month after ACS	Switch to ASA + clopidogrel if 1 month event free after index PCI	Previous DAPT	Composite of cardiovascular death, urgent revascularization, stroke, and BARC ≥2 bleeding at 1 year post ACS	13.4% vs. 26.3%, HR 0.48, 95% CI 0.34–0.68, *p *< 0.01
			**Exclusion:** History of intracranial bleeding, thrombocytopenia, long term anticoagulation				
TROPICAL-ACS [66]	2610	ACS requiring PCI and 12 months DAPT	**Inclusion:** ACS, planned treatment of prasugrel 12 months after PCI	Stepdown with 1 week prasugrel followed by 1 week clopidogrel and PFT guided maintenance therapy	DAPT with ASA + Prasugrel	Non-inferiority. Composite of cardiovascular death, myocardial infarction, stroke, or BARC ≥2 bleeding 1 year after randomization	7% vs. 9%, HR 0.81, 95% CI 0.62–1.06, *p* = 0.004 non-inferiority
			**Exclusion:** Cardiogenic shock in last 2 weeks, oral anticoagulation, indication for surgery				
POPular-Genetics [67]	2488	Primary PCI with stent	**Inclusion:** Treated MI within 12 hours of symptoms	Genetic testing determining P2Y12 inhibitor therapy	DAPT	1: NACE	1: 5.1% vs. 5.9%, AD: –0.7 percentage points, 95% CI –2.0–0.7, *p *< 0.001
			**Exclusion:** Malignancy with increase in bleeding, dialysis, severe HTN, cardiogenic shock			2: major or minor PLATO bleeding	2: 9.8% vs. 12.5%, HR 0.78, 95% CI 0.61–0.98, *p* = 0.04
TAILOR-PCI [80]	1849	Carriers of *CYP2C19* undergoing PCI for ACS or stable CAD	**Inclusion:** ACS or stable CAD	Ticagrelor DAPT in carriers	Clopidogrel DAPT in non-carriers	Composite of CV death, MI, stroke, stent thrombosis, severe recurrent ischemia at 1 year	4.0% vs. 5.9%, HR 0.66, 95% CI 0.43–1.02, *p* = 0.06
			**Exclusion:** Known CYP2C19, Cr ≥2.5, history intracranial bleeding				
HOST-REDUCE-POLYTECH ACS [65]	2338	ACS requiring PCI	**Inclusion:** Clinical ACS of ≥1 coronary lesion	low dose prasugrel + ASA after 1 month DAPT	DAPT	Composite of death, MI, stent thrombosis, repeat revascularization, stroke, BARC ≥2 bleeding at 1 year	7.2% vs. 10.1%, HR 0.70, 95% CI 0.52–0.92, *p *< 0.0001 non-inferiority
			**Exclusion:** major or active bleeding				

ACS, acute coronary syndrome; AD, absolute difference; ASA, aspirin; BARC, Bleeding Academic Research Consortium; CAD, coronary artery disease; CI, confidence interval; Cr; creatinine; CV, cardiovascular; DAPT, dual anti-platelet therapy; HR, hazard ratio; HTN, hypertension; MI, myocardial infarction; N, Number of patients; NACE, net adverse clinical events; PCI, percutaneous coronary intervention; PLATO, PLATelet inhibition and patient Outcomes.

## 3. Special Clinical Settings

Coronary artery bypass graft (CABG) surgery is usually the mainstay of therapy among patients with left main (LM) disease. However, with recent advances in intravascular imaging and coronary stent technologies, PCI has become a safe and viable alternative to surgical management [82, 83, 84]. Stenting of LM lesions are connected with an increased incidence of ischemic events and thus a prolonged DAPT duration is usually required [85, 86]. Nonetheless, a significant proportion of patients undergoing PCI for LM disease are at HBR [87]. Data on optimal DAPT in HBR patients undergoing PCI for LM disease remains sparse. A complex PCI sub-study of TWILIGHT included patients with left main disease, as well as bifurcation lesions treated with two stents, 3 vessels treated, 3 or more lesions treated, total stent length of >60 mm, atherectomy device use, surgical bypass graft or chronic total occlusion (CTO). After 3 months of standard DAPT, Ticagrelor monotherapy was shown to decrease risk of bleeding without an increase in ischemic events when compared to standard DAPT [88].

Additionally, DAPT in PCI for CTO remains limited, especially in the HBR population. However, a study looking at over 1000 patients undergoing PCI for CTO showed increased rates of death and MI among short (<12 months) vs. prolonged (≥12 months) DAPT duration [89]. Conversely, another study including 500 patients who underwent PCI for CTO showed similar ischemic and bleeding outcomes, irrespective of DAPT duration [90]. As these studies were largely underpowered, we still lack strong evidence supporting the use of a short DAPT regimen in HBR patients undergoing PCI for CTO.

## 4. Future Perspectives 

With increasing emphasis on the prognostic relevance of bleeding events after PCI, the need for studies designed to examine various antiplatelet strategies in HBR patients is paramount. Over the past few years, more studies have investigated antithrombotic therapy in HBR patients undergoing PCI. Although a lot of knowledge has been generated, there remain many unanswered questions. Indeed, the optimal duration and intensity of DAPT after PCI in HBR patients is yet to be defined. Shorter or longer DAPT durations might be beneficial in specific HBR subgroups, depending on the risk-benefit tradeoff of individual patients. In order to allow for direct comparison among RCTs, studies should adopt similar criteria to define HBR, used standardized endpoints for bleeding (BARC type 3 or 5) and ischemic events, and evaluate these two outcomes separately, whenever possible [8].

Significant work has been done with regards to the efficacy and safety of different ${\mathrm{P2Y}}_{12}$ inhibitors in the elderly population. Age 75 or greater is the most common minor HBR criteria, and therefore, recommendations for DAPT in HBR populations could apply to the elderly [8]. A TWILIGHT sub-analysis identified that in patients aged 65 or older ticagrelor monotherapy after 3 months of DAPT significantly reduced bleeding events, while preserving ischemic benefit [91]. Similarly, a sub-analysis of TICO showed that patients aged 64 or greater ticagrelor monotherapy after 3 months of DAPT decreased the rates of the primary outcome (composite of major bleeding, death, MI, stent thrombosis, stroke, or target-vessel revascularization) [92].

However, it is not understood which ${\mathrm{P2Y}}_{12}$ inhibitor is best for the elderly. Some studies have shown decreased risk of adverse events with ticagrelor when compared to clopidogrel without a concomitant increase in bleeding events [93, 94]. However, studies have also shown an increased bleeding with ticagrelor or prasugrel when compared to clopidogrel [95, 96]. Given these conflicting reports, additional randomized control trials among the elderly are required.

Ongoing studies are investigating short DAPT regimens with new stent technologies. TARGET SAFE will assess 1 month versus 6 months of DAPT among HBR patients receiving the Firehawk sirolimus eluting stent (NCT03287167) and BIOFLOW-DAPT will assess shorter DAPT among patients receiving either the new Orsiro stent compared to the Resolute Onyx stent (NCT04137510).

## 5. Conclusions

Remarkable advances in PCI technologies and techniques over the last decade have enabled more high-risk patients than ever before to undergo PCI. In particular, HBR patients constitute a third of those undergoing PCI and their management remains challenging periprocedurally and in the long-term. Due to an overlap between ischemic and bleeding risk factors in HBR patients, meticulous choice of antithrombotic therapy intensity and duration is imperative. Several bleeding avoidance strategies have recently been developed and tested in clinical trials, though only few enrolled HBR patients. Further large and well-powered studies dedicated to HBR patients are needed to establish the optimal management strategy in these vulnerable patients.
